# Yes-mediated phosphorylation of focal adhesion kinase at tyrosine 861 increases metastatic potential of prostate cancer cells

**DOI:** 10.18632/oncotarget.3391

**Published:** 2015-03-18

**Authors:** Tanushree Chatterji, Andreas S. Varkaris, Nila U. Parikh, Jian H. Song, Chien-Jui Cheng, Rebecca E. Schweppe, Stephanie Alexander, John W. Davis, Patricia Troncoso, Peter Friedl, Jian Kuang, Sue-Hwa Lin, Gary E. Gallick

**Affiliations:** ^1^ Department of Genitourinary Medical Oncology, The David Koch Center for Applied Research in Genitourinary Cancers, The University of Texas MD Anderson Cancer Center, Houston, TX, USA; ^2^ Programs in Cancer Biology and Cancer Metastasis, The University of Texas Graduate School of Biomedical Sciences at Houston, TX, USA; ^3^ Department of Pathology, College of Medicine, Taipei Medical University, Taipei, Taiwan; ^4^ Department of Pathology, Taipei Medical University Hospital, Taipei Medical University, Taipei, Taiwan; ^5^ Division of Endocrinology, Metabolism, and Diabetes, and Department of Pathology, University of Colorado Anschutz Medical Campus, University of Colorado Cancer Center, Aurora, CO, USA; ^6^ Department of Cell Biology, Radboud University Medical Center, Nijmegen, the Netherlands; ^7^ Department of Urology, The University of Texas MD Anderson Cancer Center, Houston, TX, USA; ^8^ Department of Pathology, The University of Texas MD Anderson Cancer Center, Houston, TX, USA; ^9^ Department of Experimental Therapeutics, The University of Texas MD Anderson Cancer Center, Houston, TX, USA; ^10^ Department of Translational Molecular Pathology, The University of Texas MD Anderson Cancer Center, Houston, TX, USA

**Keywords:** FAK, Yes, migration, metastasis, prostate cancer

## Abstract

To study the role of FAK signaling complexes in promoting metastatic properties of prostate cancer (PCa) cells, we selected stable, highly migratory variants, termed PC3 Mig-3 and DU145 Mig-3, from two well-characterized PCa cell lines, PC3 and DU145. These variants were not only increased migration and invasion *in vitro*, but were also more metastatic to lymph nodes following intraprostatic injection into nude mice. Both PC3 Mig-3 and DU145 Mig-3 were specifically increased in phosphorylation of FAK Y861. We therefore examined potential alterations in Src family kinases responsible for FAK phosphorylation and determined only Yes expression was increased. Overexpression of Yes in PC3 parental cells and *src−/−fyn−/−yes−/−* fibroblasts selectively increased FAK Y861 phosphorylation, and increased migration. Knockdown of Yes in PC3 Mig-3 cells decreased migration and decreased lymph node metastasis following orthotopic implantation of into nude mice. In human specimens, Yes expression was increased in lymph node metastases relative to paired primary tumors from the same patient, and increased pFAK Y861 expression in lymph node metastases correlated with poor prognosis. These results demonstrate a unique role for Yes in phosphorylation of FAK and in promoting PCa metastasis. Therefore, phosphorylated FAK Y861 and increased Yes expression may be predictive markers for PCa metastasis.

## INTRODUCTION

Increased migration of cancer cells is a key process in metastasis [1]. Alterations in the expression of numerous gene products through genetic and epigenetic changes have been shown to affect prostate cancer (PCa) cell migration. Many of these changes converge on extracellular matrix/tumor interactions that lead to signaling through Focal Adhesion Kinase (FAK), a central mediator of growth regulatory functions [2–4]. Major activators of FAK signaling include growth factor receptors and integrins, several of which are aberrantly expressed and shown to increase metastatic potential of PCa [5–9]. FAK has been shown to regulate cell survival, proliferation, angiogenesis, epithelial-to-mesenchymal transition, migration, and invasion [9–15], processes important in tumor progression and metastasis. These diverse biologic functions are mediated through the intrinsic FAK tyrosine kinase activity, as well as its role as a scaffolding protein [8, 16]. More recently, nuclear FAK has been shown to function as a co-transcription factor, increasing the expression of cyclin D1 and promoting p53 and GATA4 degradation, processes that increase cellular survival and proliferation [17, 18]. Increased expression of FAK occurs in many solid tumors, with levels of expression generally increasing with tumor progression [19–24]. In PCa, FAK is overexpressed in more than 70% of tumors relative to normal tissue [10], and increased FAK expression correlates with tumor grade, with highest expression occurring in metastases [10, 25, 26]. As FAK inhibitors have reached clinical trials [10], understanding the mechanisms by which FAK signaling pathways are activated and how FAK contributes to PCa progression and metastasis is of important clinical relevance.

FAK activation occurs as a result of a conformational change in the FAK FERM domain [7], leading to autophosphorylation at tyrosine 397. Once phosphorylated, pFAK Y397 recruits numerous signaling molecules, including Src family kinases (SFKs), through their SH-2 domains [7, 8]. Indeed, FAK was first identified as a substrate of the viral Src oncogene [27, 28]. Binding of SFKs to pFAK Y397 leads to trans-phosphorylation of multiple tyrosine sites in FAK, specifically, Y407, Y576, Y577, Y861 and Y925 [29]. Phosphorylation of these sites is responsible for “full” FAK catalytic activation as well as recruitment of multiple proteins directing signaling cascades that promote the above-described biologic functions regulated by FAK [4, 29]. Thus, FAK-SFK association is essential for most FAK functions [30–33]. In PCa, multiple SFKs are constitutively activated [34–39], and play distinct roles in PCa initiation [37, 40] and progression [34, 39, 41]. Lyn activation is involved in prostate development [37]; increases PCa proliferation [34], and increased expression of Lyn regulates androgen expression and promotes castrate-resistant PCa progression [39]. Src activation does not affect proliferation to a similar extent [34], but does promote PCa migration and metastasis [42, 43]. Fyn is highly overexpressed in PCa [35], affects hepatocyte growth factor-directed chemotaxis and is therefore important in PCa progression [44]. Mutated, constitutively activated forms of Src, Lyn and Fyn also induce different biology in primary murine prostate epithelial tissue transformed by FGF 10 [40]. Expression of activated Src kinase in these cells grown in the renal capsule led to characteristics associated with poorly differentiated, invasive adenocarcinoma; expression of activated Fyn led to PCa initiation by formation of prostate intraepithelial-like lesions and expression of activated Lyn had no obvious effect on prostate epithelial phenotypes [40]. Thus, strong evidence supports different roles of SFKs in PCa progression. Surprisingly, the effects of individual SFKs on FAK functions and how they may specifically affect PCa metastasis have not been previously examined. Further, potential roles of Yes, the closest related kinase to Src, which is also highly expressed in PCa cells [45], in promoting PCa progression, have not been examined prior to this study.

To examine potential roles of FAK-SFK complexes in mediating different properties of PCa metastasis, we used an unbiased strategy to select migratory variants of two well-studied PCa cell lines, PC3 and DU145, and examined changes in FAK phosphorylation, SFK expression and metastasis. Our results demonstrate that altered phosphorylation of FAK at a specific site, tyrosine 861, plays an important role in regulating key metastatic properties of PCa cells. We further identify a novel role of Yes in selective phosphorylation of FAK and in promoting PCa metastasis.

## RESULTS

### Isolation of highly migratory subclones of PCa cells

**Figure. 1:**
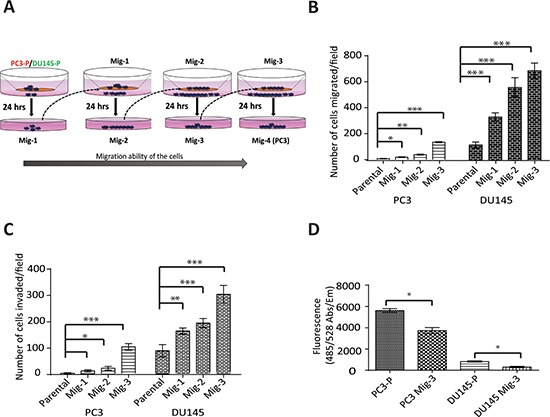
Development and characterization of highly migratory variants of PCa cells **A.** Schematic diagram of isolation of migratory variants using a modified Boyden chamber assay. **B.** *In vitro* migration and **C.** invasion after each selection was determined using the modified Boyden chamber (migration) or matrigel-coated modified Boyden chamber (invasion) for 24 hours. Migrated (or invaded) cells were counted microscopically in 5 optical fields per filter. Bars represent mean ± SD from triplicate assays. *p < 0.05, **p < 0.001, ***p < 0.0001 by Student's *t*-test. **D.** Attachment assay was performed for the indicated cells for 30 minutes. Bar graph represents mean ± SD from two independent assays performed in triplicate. *p < 0.05 by Student's *t*-test.

One of the principal roles of FAK-SFK complexes is regulating migration [4, 46, 47]. As numerous FAK activators and downstream signaling molecules are involved in this process, we developed highly migratory sublines of the prostate cancer cell lines PC3 and DU145 by multiple cycles of *in vitro* selection for cells that had migrated in a modified Boyden chamber (see schema, Fig. 1A). As described in Materials and Methods, cells that had migrated through the Boyden Chamber were grown to confluency and re-migrated. This process was repeated three times. Migratory-selected cells were termed PC3 Mig-1, PC3 Mig-2, PC3 Mig-3, DU145 Mig-1, DU145 Mig-2, and DU145 Mig-3, reflecting each cycle of selection (Fig. 1A). *In vitro* migration of these subclones was increased at each of the first three cycles of selection (Fig. 1B), with no further increases observed following subsequent selections (data not shown). The phenotype of the migratory variants has remained stable for more than 30 passages, the longest time examined. PC3 Mig-3 was increased in migration by 20 fold relative to PC3-P (PC3 parental) cells (Fig. 1B, *p* < 0.0001); DU145 Mig-3 cells were increased in migration by 6 fold (Fig. 1B) relative to DU145-P (DU145 parental) cells (*p* < 0.0001). As an independent measure of migration, time-lapse microscopy was performed for PC3-P and PC3 Mig-3 isogenic cell lines, and the average speed of the populations is plotted (Fig. S1, upper panel) along with representative images indicating the distance traveled by the cell populations in 24 hours (lower panel). Time-lapse movies of migration are shown in Videos S1 and S2. The speed of migration of PC3 Mig-3 was 0.08 ± 0.01 μm/min, compared to 0.04 ± 0.006 μm/min in PC3-P cells (*p* < 0.001). These data confirm that PC3 Mig-3 cells are more migratory than PC3-P cells.

### PC3 Mig-3 and DU145 Mig-3 cells have increased invasion, decreased attachment and decreased proliferation relative to parental cells

To investigate if the migratory selected cells were also more invasive, an *in vitro* invasion assay using a matrigel-coated Boyden chamber was performed. PC3 Mig-3 cells were increased in invasion by 25 fold relative to PC3-P cells (*p* < 0.0001); DU145 Mig-3 cells had a 4 fold increased invasion compared to DU145-P cells (*p* < 0.0001) (Fig. 1C), correlating with the increased migration in both cell models. To determine whether increased migration and invasion were due to differences in proliferation, 5 × 10^4^ PC3-P, PC3 Mig-3, DU145-P and DU145 Mig-3 were plated in a 48 well plate. Viable cells were enumerated daily for six days. The doubling times for PC3-P cells and PC3 Mig-3 cells were 19 hours and 25 hours, respectively (Fig. S2). The doubling times for DU145-P and DU145 Mig-3 cells were 19 and 24 hours, respectively (*p* < 0.05). These data are consistent with more migratory cells having reduced proliferation rates [48].

Next, the effects on cell attachment were analyzed by plating 5 × 10^4^ cells in each well of a 96-well plate and washing with PBS after 30 minutes. The number of viable cells bound to the cell culture plate was determined using Calcein AM staining. Attachment of PC3 Mig-3 cells was decreased by 33% relative to PC3-P cells (Fig. 1D, *p* < 0.05). Attachment of DU145 Mig-3 cells was decreased by 63% relative to DU145-P cells (Fig. 1D, *p* < 0.05).

### Increased expression of pFAK Y861 is associated with increased migration of PC3 Mig-3 cells

**Figure. 2:**
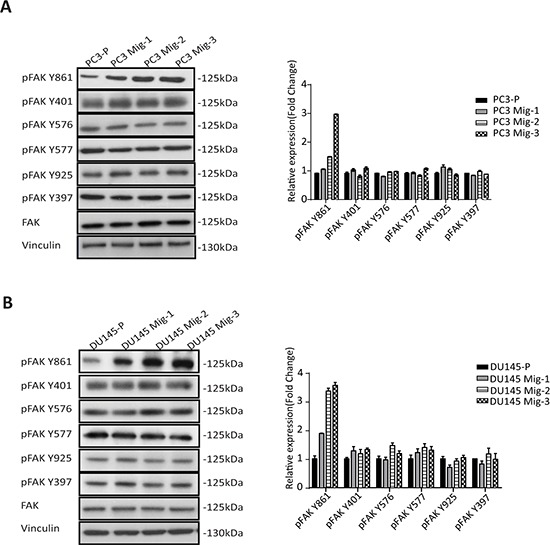
Migratory variants of PCa cells have increased phosphorylation of FAK Y861 **A.** Expression of FAK and phosphorylation of individual FAK tyrosine sites on indicated cell lysates was determined by immunoblotting (left panel). Quantification (right panel). **B.** Identical analyses were performed in DU145 cells; immunoblot (left panel); quantification (right panel).

Having established two isogenic models with increased migratory potential, we next assessed potential alterations in FAK. FAK expression and tyrosine phosphorylation at each site were determined. Expression of total FAK protein in PC3 Mig-3 (Fig. 2A) (immunoblot, left panel) and DU145 Mig-3 cells (Fig. 2B) (immunoblot, left panel) relative to the parental cells was similar. Phosphorylation of FAK Y397 (the autophosphorylation site) was not changed. However, phosphorylation of one of the SFK-dependent tyrosine sites, FAK Y861, increased with each cycle of migration selection in both PC3-P (Fig. 2A, immunoblots left panel; quantification right panel) and DU145-P cell lines (Fig. 2B, immunoblots left panel; quantification right panel), with no increase in other SFK-dependent tyrosine phosphorylation sites, i.e., FAK Y401, FAK Y577, FAK Y576 and FAK Y925.

### Met and Axl do not contribute to increased migration of PC3 Mig-3 cells

**Figure. 3:**
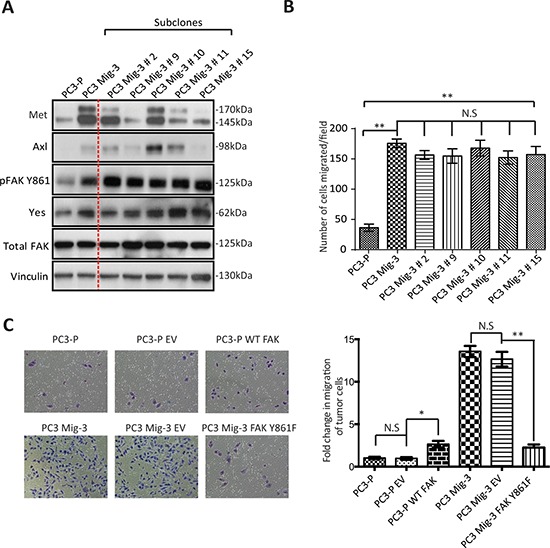
Increased phosphorylation of FAK Y861 is associated with increased migration of PC3 Mig-3 cells **A.** Expression of signaling proteins in single cell clones of PC3 Mig-3 cells. PC3 Mig-3 cells were subjected to single cell cloning; immunoblotting was performed for indicated proteins. Lane-1:PC3-P cells; lane-2:PC3 Mig-3 cells; lanes 3 to 7:subclones of PC3 Mig-3 cells. **B.** Migration assays on subclones of PC3 Mig-3 cells. Bars represent means ± SD. Three independent assays were performed in triplicate. **p < 0.001 by Student's *t*-test from three independent assays performed in triplicate; N.S, no statistical difference. **C.** Effect of overexpression of the non-phosphorylatable FAKY861F on migration of PC3 Mig-3 cells. Representative photomicrographs of migrated cells (left panel); quantification (right panel). Bars represent the means ± SD (right panel). *p < 0.01, **p < 0.001 by Student's *t*-test of three independent assays performed in triplicate.

We further examined the expression of two additional proteins, Met and Axl, which have also been shown to regulate migration and are overexpressed in prostate cancer progression [49, 50]. As shown in Fig. 3A, Met and Axl were also increased in migration-selected PC3 Mig-3 cells relative to PC3-P cells (compare lanes and 1 and 2). To examine if Axl and/or Met contributed to increased migration of PC3 Mig-3, single cell cloning of PC3 Mig-3 was performed. Expression of these proteins in five subclones is shown in Fig. 3A (lanes 3–7). Each clone consistently overexpressed pFAK Y861; however, Met and Axl were variably expressed, with some clones overexpressing either Met or Axl or both, and other clones overexpressing neither, relative to parental cells. To determine the potential contribution of Met and Axl to migration of these subclones, migration assays on each subclone were performed. As shown in Fig. 3B, all five PC3 Mig-3 subclones have increased migration relative to PC3-P cells; however, the levels of Met and Axl in these subclones did not alter *in vitro* migration. Thus, among the candidate migratory factors examined, the only consistent alteration that correlated with increased migration in all of the clones of PC3 Mig-3 cells was increased levels of pFAK Y861. Overexpression of Yes was also observed (experiments demonstrating the importance of Yes overexpression are described below). These results indicate that increased expression of pFAK Y861 is independent of Axl or Met expression and is the molecular alteration studied that is most closely associated with increased of PC3 Mig-3 cells.

To determine if the increase in the phosphorylation of FAK at Y861 is involved in the enhanced migration of PC3 Mig-3 cells, we overexpressed a non-phosphorylatable V-5 tagged form of FAK (FAKY861F) in the PC3 Mig-3 cells. Expression of this mutant is demonstrated by immunoblotting for the V-5 tag (Fig. S3A). *In vitro* migration of PC3 Mig-3 FAKY861F cells was determined in a modified Boyden Chamber assay (Fig. 3C, photomicrographs of migrated cells, left panel; quantification, right panel). Migration was reduced by 90% in PC3 Mig-3 FAKY861F cells compared to the empty vector control (*p* < 0.001). To determine whether decrease in migration in PC3 Mig-3 cells expressing FAKY861F were due to change in proliferation, growth rates were determined. As shown in Fig. S3B, proliferation rates of PC3 Mig-3 FAKY861F cells were comparable to that observed for the empty vector control cells. As an additional control, we overexpressed a V-5 tagged wild type FAK in PC3-P cells. As shown in Fig. 3C (photomicrographs, left panel; quantification right panel), FAK overexpression increased migration of PC3-P cells 3 fold, considerably less than the 20 fold increased migration observed in PC3 Mig-3 cells relative to PC3-P cells. These data indicate that phosphorylation of FAK Y861 is critical in regulating migration of PCa cells.

### Increase in yes expression and kinase activity correlates with increased migration in PC3 Mig-3 cells

**Figure. 4:**
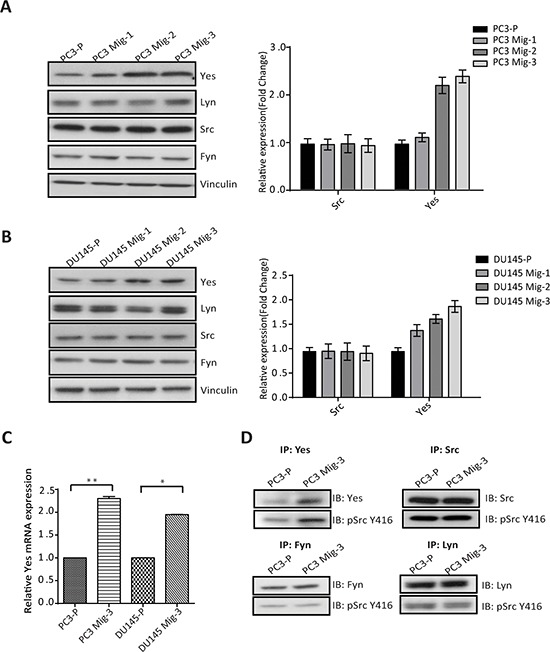
Expression and Activity of Yes in PCa cells **A.** Expression of SFKs in the migratory variants of PC3 cells was determined by immunoblotting (left panel). Quantification of expression of Yes, Lyn, Src, Fyn normalized to vinculin (right panel). **B.** Identical analyses on DU145 cells; immunoblot (left panel); quantification (right panel). Representative data are shown from three independent assays. **C.** mRNA expression of *c-yes* in indicated cells normalized to actin as a control. The data are represented as mean ± SD. **p < 0.001, *p < 0.05 by Student's *t*-test of three independent assays performed in triplicate. **D.** Activity of the SFK's was estimated by immunoprecipitation with specific antibodies for individual SFKs as indicated, followed by immunoblotting for the Src autophosphorylation site (pSrc Y416). Representative data are shown from three independent assays.

Src family kinases (SFK's) catalyze phosphorylation of all of the FAK tyrosine phosphorylation sites, excluding the autophosphorylation site (FAK Y397) [29]. Hence, we investigated the expression and activity of SFK's in PC3 Mig-3 and DU145 Mig-3 cells relative to PC3-P cells. As shown in Fig. 4A, no increased expression of Src, Fyn and Lyn was observed (immunoblot, left panel; quantification, right panel). However, a 2.5 fold increase in Yes expression was observed in PC3 Mig-3 cells relative to PC3-P cells. Similarly, Yes expression was increased by 2 fold in DU145 Mig-3 relative to DU145-P cells (Fig. 4B, immunoblot, left panel; quantification, right panel), with no increase in other Src family kinase members. As determined by qRT-PCR, c-*yes* mRNA was increased 2.3 fold (*p* < 0.001) in the PC3 Mig-3 and 2 fold (*p* < 0.05) in DU145 Mig-3 cells relative to their respective parental cells (Fig. 4C). We next examined the kinase activity of the SFKs expressed in PC3 cells. For these studies, immunoprecipitation of individual SFKs was performed using specific antibodies to each protein followed by immunoblotting with an antibody to chicken Src pY416 that recognizes the autophosphorylation sites (indicative of the activated form of the kinases) of all the SFKs examined. As shown in Fig. 4D, no increase in expression or autophosphorylation was observed for Src, Lyn and Fyn in PC3 Mig-3 cells relative to the PC3-P cells. However, Yes activity was increased by 3 fold in the more migratory PC3 Mig-3 cells relative to the PC3-P cells (Fig. 4D). These results suggest that increased expression of Yes is accompanied with increased total kinase activity in PC3 Mig-3 cells.

### Yes association with FAK is increased in PC3 Mig-3 cells

**Figure. 5:**
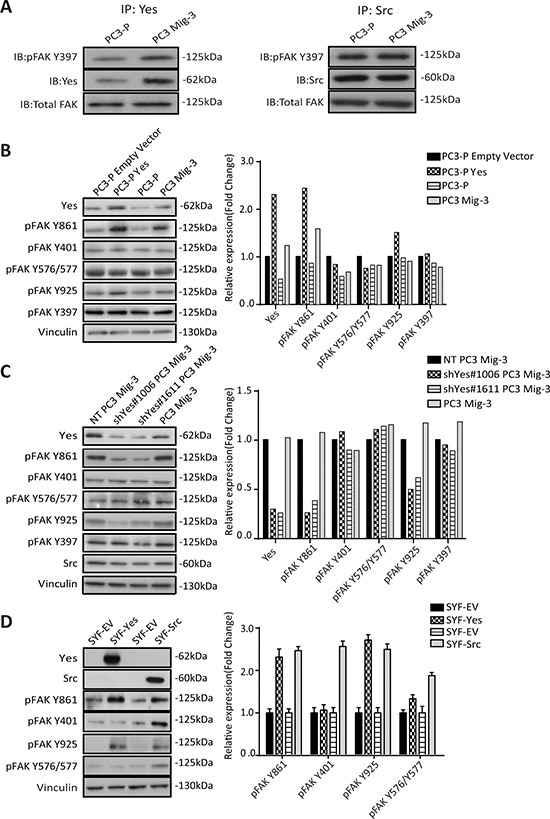
PC3 Mig-3 cells are increased in FAK association with Yes and Yes preferentially phosphorylates FAK Y861 in PC3 and SYF MEF cells **A.** FAK-Yes and FAK-Src complexes were examined by immunoprecipitating with antibodies specific to Yes (left panel) or Src (right panel), followed by immunoblotting with antibodies to pFAK 397, Yes (left panel); Src (right panel) and total FAK. **B.** Effect of Yes overexpression on FAK phosphorylation of individual FAK tyrosine sites. Empty vector was used as a control (left panel). Quantification of immunoblotting normalized to vinculin (right panel). Representative data are shown from three independent experiments. **C.** FAK phosphorylation at individual tyrosine sites was determined by immunoblotting (left panel) after silencing Yes, using two different shRNA sequences (shYes#1006 and shYes#1611). A non-targeting (NT) plasmid was used as a control. Quantification of phosphorylation of FAK at individual tyrosine sites was performed and normalized to vinculin (right panel). **D.** Phosphorylation of FAK at individual tyrosine sites by Src or Yes in *src−/−yes−/−fyn−/−* mouse embryonic fibroblasts cells was determined by immunoblotting after overexpression of Yes or Src following transfection (left panel). Quantification of phosphorylation of FAK at individual tyrosine sites normalized to vinculin (right panel).

As Yes expression and activity were increased in PC3 Mig-3 cells relative to the PC3-P cells, we next performed co-immunoprecipitation studies to determine whether there were increased Yes-FAK association in PC3 Mig-3 cells compared to the PC3-P cells. As shown in Fig. 5A (left panel), increased Yes association with pFAK Y397 was observed in the PC3 Mig-3 cells relative to the PC3-P cells (1.6 fold). In contrast, there was no obvious increase in association of Src with pFAK Y397 in PC3 Mig-3 cells relative to the PC3-P cells (Fig. 5A, right panel), indicating increased association of Yes with FAK is specific to the more migratory PC3 Mig-3 cells.

### Yes kinase preferentially phosphorylates FAK Y861 in PC3 Mig-3 cells

As Yes overexpression in the migratory variants increases Yes/FAK complexes, we next investigated the role of Yes in phosphorylation of FAK Y861. As shown in Fig. 5B, overexpression of Yes in PC3-P cells increased FAK Y861 phosphorylation by 2.4 fold, and to a lesser extent FAK Y925 phosphorylation (1.4 fold, Fig. 5B, immunoblot, left panel; quantification, right panel). Yes overexpression did not increase phosphorylation of FAK Y397, FAK Y401, FAK Y577 and FAK Y576 (Fig. 5B, immunoblot, left panel; quantification, right panel). In contrast, overexpression of Src in PC3-P cells led to an increased phosphorylation of all SFK sites (Fig. S3C).

In a second approach to determine if Yes preferentially phosphorylated FAK Y861, Yes was silenced in PC3 Mig-3 and DU145 Mig-3 cell lines cells using two pLKO.1 vectors directing the expression of Yes specific shRNA sequences. In PC3 Mig-3 cells, knockdown of Yes led to decreased expression of pFAK Y861 (4 fold and 2.6 fold) and to a lesser extent pFAK Y925 (2 fold and 1.6 fold), with no significant effect on phosphorylation of the other FAK tyrosine residues, as shown in Fig. 5C (immunoblot, left panel; quantification, right panel). In contrast, silencing Src led to decreased phosphorylation of all the SFK phosphorylated FAK tyrosine residues (Fig. S3D). Similarly, silencing Yes in DU145 Mig-3 cells lead to decreased phosphorylation of FAK Y861 (Fig. S4A, immunoblotting, left panel; quantification, right panel). While the above experiments provided strong evidence that Yes kinase preferentially phosphorylates FAK Y861 relative to the other FAK tyrosine sites, these experiments could not exclude potential roles of other SFKs expressed in PCa cells. Therefore, to further determine whether Yes is restricted in its ability to phosphorylate FAK, Yes or Src were transiently overexpressed in SYF (*src*−/−*yes*−/−*fyn*−/−) mouse embryo fibroblasts. Overexpression of Src (alone) in the SYF cells led to increased phosphorylation of all the SFK-dependent tyrosine phosphorylation sites (Fig. 5D). However, overexpression of Yes led to a 2.3 fold increase in phosphorylation of FAK Y861 with a minor increase in phosphorylation of FAK Y925 (Fig. 5D, immunoblot, left panel; quantification, right panel); no other SFK sites were appreciably phosphorylated. These data are consistent with a novel and restricted role of Yes in preferentially phosphorylating FAK Y861, and to a lesser extent, FAK Y925.

### Yes expression affects migration of PCa cells

**Figure. 6:**
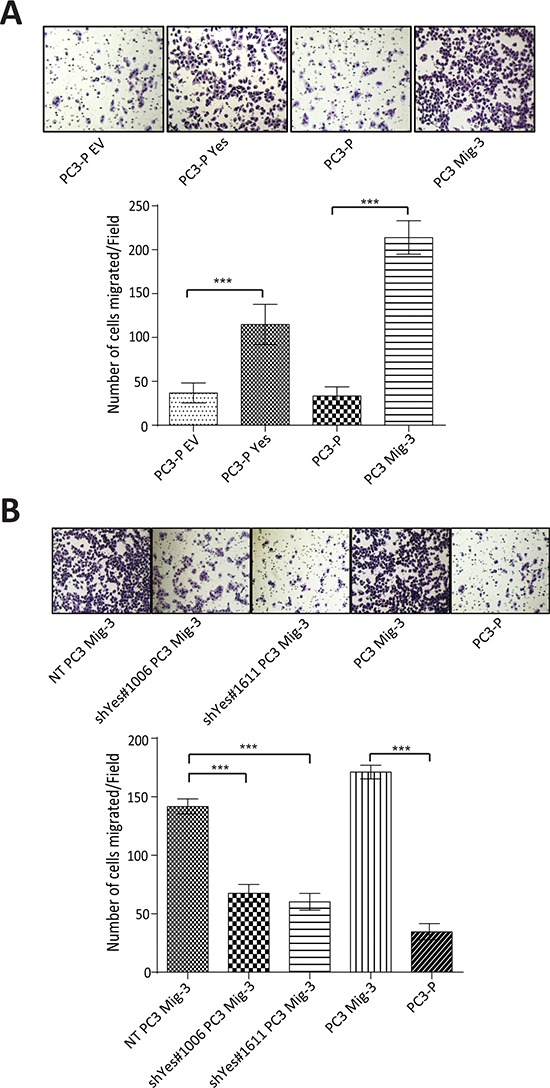
Yes regulates migration of PC3 cells **A.** Effect of overexpression of Yes on migration of PC3-P cells. Representative photomicrographs of migrated cells (upper panel). Bars represent means ± SD (lower panel). ***p < 0.0001 by Student's *t*-test of three independent assays performed in triplicate. **B.** Effect of silencing of Yes in PC3 Mig-3 cells on migration. Representative photomicrographs of migrated cells (upper panel). Quantification of migration (lower panel); bars represent mean ± SD (lower panel). ***p < 0.0001 by Student's *t*-test of three independent assays were performed in triplicate.

The above results suggested that increased Yes expression and activity were responsible for the observed effects on FAK Y861 phosphorylation. To examine if increased Yes expression directly affected PCa cell migration, we performed migration assays in PC3-P cells, PC3-P cells overexpressing Yes and in PC3 Mig-3 and DU145 Mig-3 cells in which Yes was silenced. Overexpression of Yes in PC3-P cells led to a 3.4 fold (*p* < 0.0001) increase in migration (Fig. 6A, photomicrographs of migrated cells, upper panel; quantification, lower panel), while knockdown of Yes in PC3 Mig-3 cells led to a 50% (shYes#1006, *p* < 0.0001) and 57% (shYes#1611, *p* < 0.0001) reduction in migration relative to non-targeting PC3 Mig-3 control cells (Fig. 6B, photomicrographs of migrated cells, upper panel; quantification, lower panel). Similarly, knockdown of Yes in DU145 Mig-3 cells led to a 94% (shYes#1006, *p* < 0.0001) and 91% (shYes#1611, *p* < 0.0001) reduction in migration relative to the non-targeting virus-infected DU145 Mig-3 cells (Fig. S4B). Collectively, these data demonstrate that Yes phosphorylation of FAK Y861 corresponds with increased migration of both migratory variants, PC3 Mig-3 and DU145 Mig-3.

### PC3 Mig-3 cells induce increased lymph node metastases relative to PC3-P cells in orthotopic nude mouse models

**Figure. 7:**
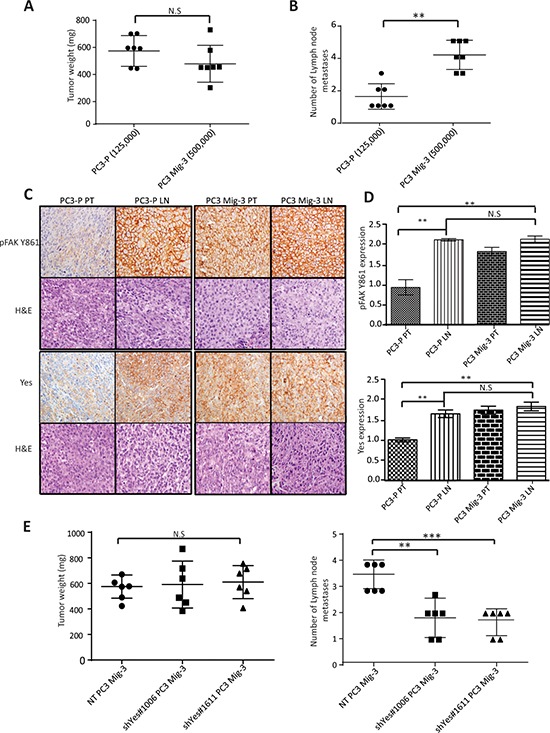
Increased phosphorylation of FAK Y861 and increased expression of Yes promotes lymph node metastasis of PC3 Mig-3 **A.** Weights of PC3-P and PC3 Mig-3 tumors following intraprostatic injection of 125,000 PC3-P cells and 500, 000 PC3 Mig-3 cells (6 mice/group). Graph represents average tumor weight ± SEM from PC3-P and PC3 Mig-3 cells. N.S-no statistical difference. **B.** Number of lymph node metastases in the above-described experiment is represented as mean ± SEM. **p < 0.001 by Tukey's test. **C.** Expression of pFAK Y861 and Yes in fixed sections of primary tumors and lymph node metastasis from PC3-P and PC3 Mig-3 as determined by immunohistochemistry (left panel); corresponding H&E are shown below. **D.** Quantification of staining- pFAK Y861 (top panel), Yes (bottom panel). Bars represent average intensity ± SD. **p < 0.001 by Student's *t*-test. **D.** Effect of Yes silencing on lymph node metastasis of PC3 Mig-3 cells (6 mice/group). Graph represents average tumor weight ± SEM. N.S-no statistical difference. **E.** Number of lymph node metastasis represented as mean ± SEM. **p < 0.001, ***p < 0.0001 by Tukey's test.

As the selected migratory cells demonstrated several properties associated with increased metastasis, we investigated the metastatic potential of PC3 Mig-3 cells by inoculating cells into the prostate of nude mice (the orthotopic site) as described in Materials & Methods. Mice were sacrificed after 4 weeks. All mice developed primary tumors (see Table 1). In agreement with *in vitro* growth rates, PC3 Mig-3 tumors at similar inocula were smaller in size (*p* < 0.001) (Fig. S5A) and had fewer Ki67 positive cells relative to the PC3-P (*p* < 0.05) (Fig. S5B; photomicrograph of Ki67 positive cells, upper panel; quantification, lower panel). Lymph node metastases (orthotopic injection models lead to lymph node, but not distant metastases) were assessed by identifying solid, opaque and enlarged iliac lymph nodes. An example of a tumor-positive lymph node for both PC3-P and PC3 Mig-3 cells before complete dissection is shown in Fig. S6A. Insets show examples of enlarged nodes with white appearance (20X magnification). Representative primary tumors and lymph node metastases from five mice after complete dissection are shown in in Fig. S6B. Presence of tumor cells in the node was histologically confirmed using H&E staining as indicated in photomicrographs of representative lymph node metastases shown in Fig. S7A, and S7B, left panels. Despite significantly smaller primary tumors obtained from 125,000 PC3 Mig-3 cells relative to 125,000 PC3-P cells (*p* < 0.05), the number of lymph node metastases was increased in mice injected with PC3 Mig-3 cells (*p* < 0.05) (Table 1). To obtain similar-sized primary tumors from PC3-P and PC3 Mig-3 to most accurately compare development of lymph node metastases, we increased the inocula of PC3 Mig-3 tumor cells implanted intraprostatically. An initial inoculum of 125,000 PC3-P cells and 500,000 PC3 Mig-3 cells gave statistically similar primary tumor sizes (See Fig. 7A, Table 1 and Fig. S6B). The average number of lymph node metastases when primary tumors were of similar size was 4.1 ± 0.3 in the PC3 Mig-3 group relative to 1.5 ± 0.3 lymph node metastases in PC3-P tumors (*p* < 0.001) (Fig. 7B). These data demonstrate that PC3 Mig-3 cells are more metastatic relative to the PC3-P cells.

**Table 1:** Average weight of primary tumor and lymph node metastasis following orthotopic injection of PC3-P and PC3 Mig-3 cells

Group	Average Tumor wt. (mg)/range	Incidence of primary tumor	Average number of LN mets/range	Incidence of LN mets
PC3-P (125,000)	598.5 (220–875)	9/9	1.8(1–3)	9/9
PC3 Mig-3 (125,000)	183.1 (45–349)*	7/7	2.7(2–4)*	7/7
PC3 Mig-3 (250,000)	339.5 (204–490)	7/7	2.6(1–3)*	7/7
PC3 Mig-3 (500,000)	423.5 (303–730)	7/7	4.1(3–5)*	7/7

Abbreviation LN mets: Lymph node metastases

* *p* < 0.05 by Tukey's test compared to PC3-P (125, 000)

### Increased expressions of pFAK Y861 and Yes kinase are associated with PCa lymph node metastases in an orthotopic nude mouse model

As discussed above, PC3-P cells are also able to metastasize to the lymph node following orthotopic injection, albeit to a lesser extent. We therefore determined if the lymph node metastases from PC3-P also exhibited increased phosphorylation of FAK Y861 and increased expression of Yes kinase. pFAK Y861 and Yes expression in the primary tumors and lymph node metastases was assessed by immunohistochemistry. Quantification of the staining was performed after randomly selecting areas from the whole tumor scans from the primary tumors and lymph node metastases. Examples of lymph nodes stained for pFAK Y861 are shown in Fig. S7A and S7B (right panels). Magnified random sections (20X, insets) indicate examples of representative areas used in quantification. As expected, in PC3 Mig-3 primary tumors, pFAK Y861 expression was increased 2.5 fold relative to PC3-P primary tumors (*p* < 0.001) (Fig. 7C, compare panel 3 to panel 1, quantification Fig. 7D top panel). However, levels of pFAK Y861 in the lymph node metastases from PC3-P cells were also increased relative to primary tumors, to levels similar to those observed in PC3 Mig-3 primary tumors and lymph node metastases (Fig. 7C, compare panel 2 to panels 3 and 4, quantification Fig. 7D top panel). These results demonstrate that lymph node metastases are associated with increased pFAK Y861. We next examined Yes expression in PC3-P primary tumors and lymph node metastases. Similar to what was observed for pFAK Y861, Yes expression was increased by 2.3 fold in PC3-P lymph node metastases compared to PC3-P primary tumors (*p* < 0.001) (Fig. 7C, compare panel 1 to panel 3) with no further increase in expression of Yes in PC3 Mig-3 lymph node metastasis relative to PC3 Mig-3 primary tumor (Fig. 7C; compare panel 3 to panel 4; quantification of IHC, Fig. 7D bottom panel). Additionally, Yes expression in the lymph node metastasis relative to the primary tumors was also determined at the RNA levels using qRT-PCR. Lymph node metastases from PC3-P tumors were increased in expression of Yes relative to the primary tumors (Fig. S8). These observations suggest that increased phosphorylation of FAK Y861 and increased expression of Yes are associated with PCa lymph node metastases.

### Yes promotes PCa lymph node metastasis in the orthotopic nude mouse model

The above studies suggested that increased Yes expression might be responsible for the observed increase in lymph node metastases in our migratory-selected variants. Therefore, PC3 Mig-3 cells in which Yes was silenced were examined for the development of lymph node metastases after orthotopic implantation into the prostates of nude mice. For these experiments, Non-targeting control (NT) and Yes-silenced cell lines with two different sequences were transduced with a plasmid directing luciferase expression. Following orthotopic injections, mice were sacrificed when the primary tumors reached similar sizes (Table 2, Fig. 7E, left panel). Lymph node metastases were formed in all the groups (Table 2). However, decreased Yes expression decreased development of lymph node metastases. Control NT PC3 Mig-3 cells formed 3.5 ± 0.2 lymph node metastasis compared to 1.8 ± 0.3 lymph node metastases in the shYes#1006 group (*p* < 0.001) and 1.6 ± 0.2 lymph node metastases in the shYes#1611 group (*p* < 0.0001) (Fig. 7E, right panel). Therefore, silencing Yes in PC3 Mig-3 cells decreased their ability to metastasize to lymph nodes, indicating that Yes is important to this metastatic process.

**Table 2:** Average weight of primary tumor and lymph node metastasis following orthotopic injection of NT PC3 Mig-3, shYes#1006 PC3 Mig-3 and shYes#1611 PC3 Mig-3 cells

Group	Average Tumor wt. (mg)/range	Incidence of primary tumor	Average number of LN mets/range	Incidence of LN mets
NT PC3 Mig-3	556.7 (490–670)	6/6	3.5 (3–4)	6/6
shYes #1006 PC3 Mig-3	572.8 (436–694)	6/6	1.8 (1–3)**	6/6
shYes #1611PC3 Mig-3	604.7 (494–687)	6/6	1.6 (1–3)**	6/6

Abbreviation LN mets: Lymph node metastasis

** *p* < 0.001 by Tukey's test relative to NT PC3 Mig-3

### Yes and pFAK Y861 expression in human prostate cancer specimens

**Figure. 8:**
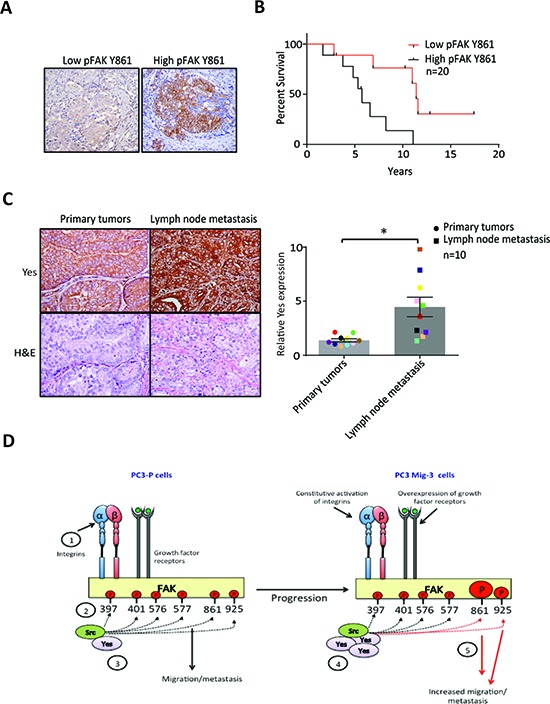
Expression of pFAK Y861 and Yes in PCa patients **A.** Expression of pFAK Y861 in lymph node metastasis from patient tumors by immunohistochemistry. **B.** Kaplan-Meier survival plot. Average survival time of cases with high pFAK Y861 expression: 6.1 ± 1 years with low pFAK Y861: 11.7 ± 1.7 years; *p* = 0.008 by log rank test. **C.** Expression of Yes in paired primary tumors and lymph node metastases from prostate cancer patients by immunohistochemistry. Representative photomicrographs in primary tumor (upper right panel) and corresponding lymph node metastasis (upper left panel). Corresponding H&E are shown in lower panels. Bars represent the average fold-change ± SEM in Yes expression in primary tumor compared to corresponding lymph node metastases, with matching colored data points indicating primary tumor and corresponding lymph node metastasis (right panel). *p < 0.05 by Student's *t*-test. **D.** Model for restrictive phosphorylation of FAK Y861 and FAK Y925 by Yes in the PC3 Mig-3 cells. 1) Integrin clustering and/or growth factor receptor activation lead to phosphorylation of FAK Y397. 2) pFAK Y397 recruits SFKs via their SH2 domain. 3) SFKs phosphorylate FAK at specific tyrosines, recruiting signaling proteins important to FAK's scaffolding function. 4) PCa progression leads to increased transcription and expression of Yes, leading to preferential binding of Yes to pFAK Y397. 5) Yes binding to pFAK Y397 leads to preferential phosphorylation of FAK Y861 and to a lesser extent, FAK Y925 increasing migration, invasion and metastatic potential of PCa cells.

We next examined pFAK Y861 expression in lymph node metastases of prostate cancer patients using immunohistochemistry. Phosphorylated FAK Y861 was observed in tumor-positive lymph nodes in 50% of specimens (*n* = 20). Representative images of lymph node metastases are shown in Fig. 8A. Patients with positive or high pFAK Y861 expression had an overall survival of 6.1 ± 1.0 years. In contrast, patients with negative or low pFAK Y861 expression had an overall survival of 11.7 ± 1.7 years, (*p* = 0.008) (Fig. 8B). These data indicate that expression of pFAK Y861 in prostate cancer patients correlates with decreased survival.

Next, we examined Yes protein expression in PCa primary tumors and matching lymph node metastases (*n* = 10) by immunohistochemistry. Representative photomicrographs of a primary tumor and corresponding lymph node metastasis are shown in Fig. 8C (left panel). Yes expression in every lymph node metastasis was significantly increased relative to its corresponding primary tumor (*p* < 0.05) as indicated by the matching colors for primary tumor and corresponding lymph node metastasis in the graph (Fig. 8C, right panel). Yes expression was increased by an average of 3.2 fold in the lymph node metastases relative to the primary tumors (*p* < 0.05) (Fig. 8C). In addition to detection in PCa cells, high Yes expression was observed in lymphocytes. Lymphocyte expression of Yes was excluded in quantitation of changes in tumor cells. Therefore, both Yes and its principal FAK target (pY861) are increased in lymph node metastasis, suggesting their importance in PCa metastasis.

## DISCUSSION

We demonstrate that overexpression of Yes in our model migratory cell lines increases Yes/FAK association, which is responsible for increased phosphorylation of FAK Y861, leading to increased cell migration *in vitro* and metastasis *in vivo* (see model Fig. 8D). FAK is a mediator of numerous biologic properties that when deregulated are associated with increased metastatic potential [51, 52]. Additionally, many of the proteins in signaling cascades activated through the adaptor functions of FAK are aberrantly expressed in cancer cells, several of which also induce increased metastatic potential [9, 10, 52, 53]. In prostate cancer, FAK is overexpressed, with overexpression correlating with higher tumor grade and metastasis [25, 26]. Overexpression of numerous growth factor receptors [54–57] and constitutive activation of integrins [56] and activators of integrins [58] in prostate cancer increase FAK functions and increase metastasis in immunodeficient mouse models. Autophosphorylation of FAK leads to association with multiple Src family kinases [43, 59], which are themselves constitutively activated in progressive stages of PCa [34, 45], and are responsible for phosphorylation of tyrosine residues on FAK that derepress FAK adaptor functions leading to aberrant activation of signaling cascades that increase metastatic potential. In prostate cancer cells, increased FAK-Src signaling is associated with migration of PC3 and DU145 PCa cells [31], but the roles of phosphorylation of each of the individual FAK sites and the signaling cascades activated were not examined previously.

Numerous studies have analyzed the complexes associated with FAK tyrosine phosphorylation sites, and the functions that they mediate [60–63]. Yet, very few studies have examined if alterations in FAK phosphorylation might contribute to FAK functions associated with metastasis. Lim *et al*. demonstrated that phosphorylation of FAK at Y861 was important to Ras transformation of fibroblasts [60], and increased Src activity was associated with increased FAK phosphorylation and increased migration in tamoxifen-resistant MCF breast cancer cells [64]. However, the mechanisms by which these altered phosphorylations occur and their potential roles in metastatic capability are unknown.

Therefore, to better unravel roles of altered FAK regulation in promoting migration, a key property in metastasis, we established stable isogenic models of prostate cancer cell lines with increased migration through a modified Boyden Chamber selection assay. In both PC3 and DU145 cells selected by this strategy, a signature alteration associated with increased migration of cells is increased phosphorylation of FAK Y861. We asked whether there were changes in expression/activity of SFKs that might account for the altered FAK phosphorylation. We demonstrate, for the first time, that Yes phosphorylates only FAK Y861 and FAK Y925, unlike Src, which directs phosphorylation of each of the known phosphorylated FAK tyrosine residues besides Y397. These results demonstrate that different Src family kinases have distinct abilities in phosphorylating FAK sites that mediate signaling cascades associated with metastasis. Interestingly, it was reported that overlapping downstream signaling pathways of FAK Y861 and FAK Y925 can compensate for one another in mediating migration of cells via recruitment of p130Cas, formation of complexes with Crk/DOCK180 and activation of Rac GTPases, promoting lamelipodia and invadopidia formation [60, 62]. This functional overlap may explain why pFAK Y925 expression was not increased in our migratory selected isogenic variants. As Src is highly activated in these prostate cancer cell lines [34], it may be that Src phosphorylation of FAK Y925 is sufficiently robust such that Yes cannot further increase FAK Y925 phosphorylation; hence increased FAK Y925 phosphorylation was not observed in our selected cells. We did not detect an increased expression of FAK, which was reported to be associated with more progressed stages of PCa [25, 26]. We speculate that at least one role of increased expression of FAK observed in progressive stages of PCa might be to provide more tyrosine-phosphorylated docking sites for proteins involved in signaling cascades that mediate metastatic potential. As FAK is also a co-transcription factor directing degradation of p53 [17] and expression of cyclin D1 [18], and leads to degradation of GATA 4 and p53, another possible role for overexpression of FAK is its nuclear function that leads to, among other properties, increased proliferation. Further experiments are required to resolve these possibilities.

To begin to address the mechanism by which Yes is upregulated in highly migratory cells, a cDNA array analysis was done comparing mRNA expression between PC3-P and PC3 Mig-3 cells (collaboration with Dr. David McConkey and Dr. Woonyoung Choi at UT MD Anderson Cancer Center; Houston; TX). Several transcription factors, including *c-MYC, FOXA1* and *HEY1* with binding sites on the Yes promoter region were overexpressed in the migratory variants (data not shown), suggesting a possible mechanism for Yes overexpression that will require further testing.

To examine whether the observations in our model system are clinically relevant, we assessed Yes and pFAK Y861 expression in human specimens from PCa lymph node metastasis compared to the corresponding primary tumor by immunohistochemistry. Our results demonstrated that pFAK Y861 was not only increased in a subset of lymph node metastases, its increase corresponded with poorer patient prognosis. Further, in patients in which paired primary tumors and lymph node metastases could be obtained, Yes expression was also increased. Increases in Yes expression observed in our immunohistochemical analysis were similar to increases in Yes mRNA expression observed by Varambally *et al*. [65], although in that study statistical significance was not achieved likely due to insufficient number of tissues examined. These data support the biological relevance of the model systems we developed, and implicate increased Yes expression and pFAK Y861 expression as potential prognostic markers. Although FAK can be phosphorylated by multiple Src family kinases, the striking increase of pFAK Y861 in lymph node metastases and its correlation with poor prognosis would suggest that high expression of FAK Y861 in primary tumors might be an especially useful marker for more aggressive treatment, as when patients with primary tumors should undergo “active surveillance” versus treatment is often uncertain. Whether pFAK Y861 should be used in conjunction with high Yes expression in this regard requires analysis of many more specimens. Finally, lymph node metastases were examined specifically as bone metastases are not observed in our orthotopic model. Future studies are planned to determine if increased Yes expression and its ability to increase FAK Y861 phosphorylation play a role in development of bone metastases.

In summary, we demonstrated the importance of phosphorylation of FAK Y861 in metastasis of PCa and further identified differences in the ability of SFKs to phosphorylate FAK, with Yes restricted to phosphorylating only FAK Y861 and FAK Y925. Our results demonstrate a heretofore-unknown role of Yes in promoting PCa metastasis, as well as the importance of pFAK Y861 in regulation of PCa migration.

## MATERIALS AND METHODS

### Cell culture

The PC3 cell line was a gift from the laboratory of Dr. Isaiah J. Fidler, and was maintained in DMEM F-12 medium (Hyclone, USA) supplemented with 10% FBS (Hyclone, USA). The DU145 cell line was a gift from the laboratory of Dr. Renata Pasqualini and was maintained in RPMI 1640 (Corning CellGro, USA) supplemented with 10% FBS (Hyclone, USA). SYF (*src−/− yes−/− fyn−/−*) mouse embryonic fibroblasts were obtained from the American Type Tissue Culture Collection, Manassas, VA, USA) and grown in DMEM media containing 10% FBS, glutamine and pyruvate. Cells were checked every six months for *Mycoplasma* contamination (MycoAlert™ Mycoplasma detection kit, Lonza, USA), and found to be mycoplasma-free. Identity of cells was confirmed by fingerprinting analysis by the MD Anderson Cancer Center Department of Systems Biology, Core Facility.

### Migration and invasion assays

Migration and Invasion abilities of the PC3 and DU145 cells were determined by the modified Boyden chamber migration assay as described by Lesslie *et al*. [66].

### Time-lapse microscopy and quantification of cell migration

Subconfluent tumor cells were detached with 2 mM EDTA (Ambion #AM9260G), embedded (33,000/100 μl) in 3D fibrillar collagen lattices (PureCol, Advanced BioMatrix, Catlog #5005-B; final concentration 1.7 mg/ml). To construct the migration chamber, an object slide and a coverslip were connected by a spacer composed of vaseline / paraffin (1:1), resulting in an approximate chamber size of 20 × 20 × 0.5 mm and a volume of ~200 μl. After addition of medium, spontaneous migration was monitored by digital time-lapse, bright-field inverse microscopy (air objectives, 10x, NA 0.20; Leica) at 37°C using CCD cameras (Sentech, USA) and the 16-channel frame grabber software (Vistek, CA) for 24 hour with 4-min frame intervals. Time-resolved population speed was obtained by single-cell tracking (Autozell 1.0 software; Centre for Computing and Communication Technologies [TZI], University of Bremen, Bremen, Germany) of xy paths with 12-min step intervals (tumor cells). The average speed per cell was calculated from the length of the path divided by time, including “go” and “stop” phases.

### Cell attachment assays

PC3 and DU145 cells (5 × 10^4^ cells/100 μL) were seeded into each well of a 96 well plate and were incubated for 30 minutes at 37°C after which the wells were washed with PBS three times and incubated with 1 μmol/L Calcein AM (Invitrogen Life Technologies, USA) for 3 minutes. The cells that attached to the plate were quantified by measuring the fluorescence intensity at 458/528 nm in each well on a Synergy HT fluorescent plate reader (BioTeK, USA). All experiments were performed in triplicate.

### Proliferation assays

Cells were seeded (5 × 10^3^ cells/well) in 6-well tissue culture dishes. At 24 hour intervals, the media was removed, cells were trypsinized using TrypLE dissociation reagent (Gibco, USA). The cell suspension (500 μl) was counted using an automated cell viability analyzer Vi-Cell XR (Beckman Coulter, USA). All experiments were performed in triplicate.

### Immunoblotting and immunoprecipitation

Immunoblotting was performed as described by Summy *et al.* [67]; immunoprecipitation as described by Windham *et al.* [68]. Antibodies used in the studies are shown in Table S1.

### RNA isolation and quantitative real-time RT-PCR (qRT-PCR)

RNA was isolated from the cells using RNAeasy™ mini kit (Catalog # 74104, Qiagen, USA), as described by Varkaris *et al*. [69]. The primer sequences for Yes were forward: 5′-TCCTGCTGGTTTAACAGGTGGTG-3′ and reverse: 5′-TGCTTCCCACCAATCTCCTTCC-3′.

### Lentiviral-mediated FAKY861F expression

The FAK Y861F and empty vector plasmids were constructed using the pLenti6/V5 plasmid from Invitrogen, USA. These plasmids contain a blasticidin-resistance gene and a gene encoding V5 tagged mutant FAK Y861F. PC3 Mig-3 cells were infected with the lentivirus in the presence of 4 μg/mL polybrene (Sigma, USA). After 24 hours of infection, the media was replaced with DMEM F-12 and RPMI 1640 containing 10 μg/ml Blasticidin Hcl.

### Lentivirus-mediated Yes and Src silencing

Mission shRNA bacterial glycerol stock plasmids for Yes were purchased from Sigma-Aldrich. Sequences used for Yes were TRCN0000001611: CCGGACCACGAAAGTAGCAATCAAACTCGAGTT TGATTGCTACTTTCGTGGTTTTTT and TRCN00000 10006: CCGGTGGTTATATCCCGAGCAATTACTCG AGTAATTGCTCGGGATATAACCATTTTT. A non-targeting control from Sigma (Cat.#SHC016): CCGGGCGCGATAGCGCTAATAATTTCTCGAGAAA TTATTAGCGCTATCGCGCTTTT was used along with the shRNA plasmid. For silencing Src, ready-to-transfect short hairpin (sh) RNA-GFP-puromycin constructs against Src (#SR304574) were purchased from OriGene Technologies, USA. A universal scrambled negative control shRNA (#SR30004) was provided by the manufacturer. For lentivirus production (shYes plasmids), the pLKO.1 plasmid (3 μg) was co-transfected with the packaging plasmid pCMV-dR8.2 dvpr (3 μg) and the envelope plasmid pCMV-VSV-G (0.6 μg) in a ratio of 5:5:1 into 293FT cells in one 100-mm plate (Life Technologies, USA) using Lipofectamine™ 2000 (Life Technologies, USA) as describer by Jin *et al*. [58]. The medium was changed after 24 hours and replaced again with 5 μg/ml puromycin after 48 hours and incubated for one week to select stable cells in which expression of Yes was silenced.

### Yes and Src overexpression

Yes was transiently overexpressed in the PC3-P cells using the pCMV6-XL5 Yes expression plasmid (Catalog # SC116734, OriGene Technologies, Inc., USA). Empty vector control was provided by the manufacturer (Catalog #pCMV6-XL5). The Yes plasmid was sequenced using VP1.5 (forward) 5′ GGACTTTCCAAAATGTCG 3′ and XL39 (reverse) 5′ ATTAGGACAAGGCTGGTGGG 3′. For overexpression of Src, the Src sequence was excised and cloned into the *Hind*III/*Bam*HI cloning site of pCDNAIII (Invitrogen, USA), thus allowing for selection of G418-resistant clones as described by Windham *et al.* [68]. This plasmid was transfected into PC3 cells and SYF MEFs using jetPRIME™ (Polyplus-transfection, USA) according to manufacturer's instructions.

### Immunohistochemical staining and quantification

Immunohistochemistry was performed as described by Jin *et al*. [58] and quantified as described by Park *et al*. [34]. Briefly, stained primary tumors and lymph node metastases we scanned under 10X magnification. Five representative images were taken at random and brown colored images specific for DAB staining were extracted by the color deconvolution macro, inversed, and measured for intensity using NIH imageJ internal commands, as we and others have published previously. pFAK Y861 staining in human specimens were scored as positive if > 10% of cells were reactive and negative if < 10% of cells were reactive.

### *In vivo* tumorigenicity assay

PC3-P and PC3 Mig-3 cells were detached from subconfluent cultures, centrifuged and resuspended in Ca2^+^-free and Mg2^+^-free HBSS (Life Technologies, USA). Intraprostatic injections were performed as described by Park *et al*. [70]. Male athymic nude mice (Ncr *nu/nu*; ages 8–12 weeks; the National Cancer Institute-Fredrick Animal Production Area, USA) were anesthetized with pentobarbital sodium i.p (0.5 mg/1 gm of body weight; Nembutal (Abbott laboratories, USA) and placed in a supine position. A midline incision was made in the lower abdomen and the prostate was exteriorized. 25 μL of HBSS containing 125,000 PC3-P cells or (1.25 × 10^5^, 2.5 × 10^5^, 5 × 10^5^ or 1 × 10^6^) PC3 Mig-3 cells were injected to the dorsolateral side of the prostate. The incision was closed with surgical metal clips (Braintree Scientific, Inc, USA).

### Statistical analyses

ANOVA and Tukey's test was conducted to compare differences in tumor weight and incidence of lymph node metastasis. Mann-Whitney *U*-test was conducted to compare differences in speed of migration using time-lapse microscopy. Survival analysis was performed using Kaplan-Meier curves and statistical significance was assessed by Log-rank's test. Unpaired Student's *t*-test was conducted to compare the differences in all other assays. All the statistical analyses were performed with the GraphPad Prism software (version 6; GraphPad Software, USA).

## SUPPLEMENTARY FIGURES AND TABLES


