# Supplementary material for: Yes-mediated phosphorylation of focal adhesion kinase at tyrosine 861 increases metastatic potential of prostate cancer cells
Source: Oncotarget. 2015 Mar 18;6(12):10175–94. doi: 10.18632/oncotarget.3391 (PMC4496348; doi:10.18632/oncotarget.3391)
Supplement: Supplementary file 1 [file oncotarget-06-10175-s001.pdf]

SUPPLEMENTARY FIGURES AND TABLES

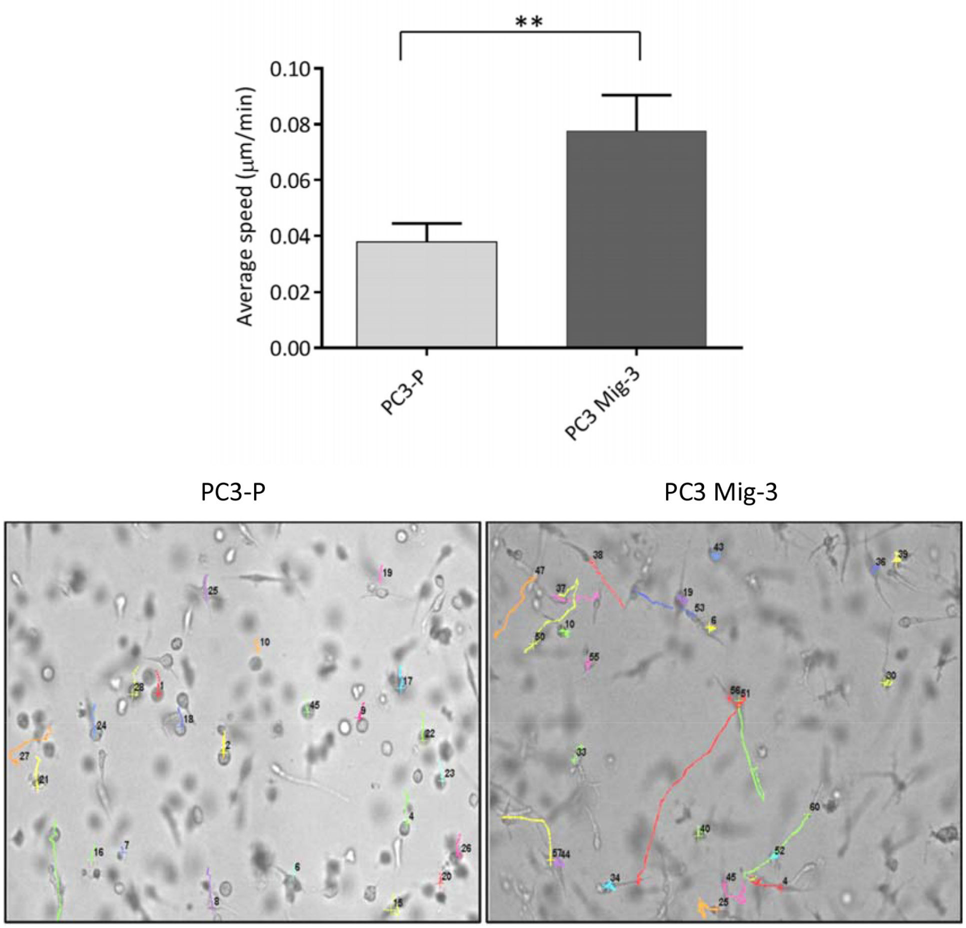

Supplementary Figure S1: Migration of PC3 Mig-3 and DU145 Mig-3 cells. Spontaneous migration was recorded using time-lapse microscopy at 10X magnification as described in Materials and Methods. Time-resolved population speed was obtained by single-cell tracking using the Auto Zell software. Bars represent mean ± SEM (upper panel). $**p < 0.001$ by Mann-Whitney *U*-test. Representative photomicrographs indicating the tracks of distance traveled by PC3-P (PC3-parental) cells and PC3 Mig-3 cells in 24 hours (lower panel).

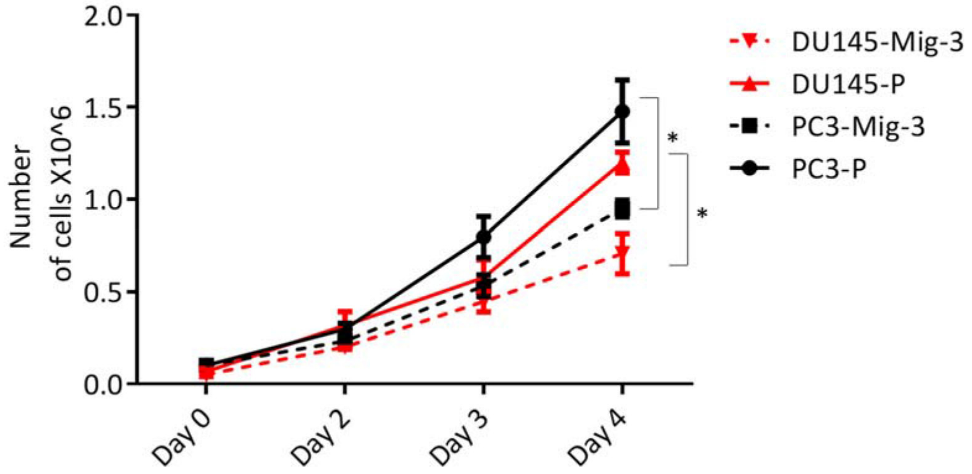

Supplementary Figure S2: Proliferation of PC3 Mig-3 and DU145 Mig-3 cells. Growth rates of PC3 and DU145 cells were determined after plating 5×10^3 of indicated cells, followed by trypsinization, trypan blue staining and counting cells using a Coulter counter after indicated times.*$p < 0.05$ by Student's *t*-test. Representative data are shown from three independent assays, performed in triplicate.

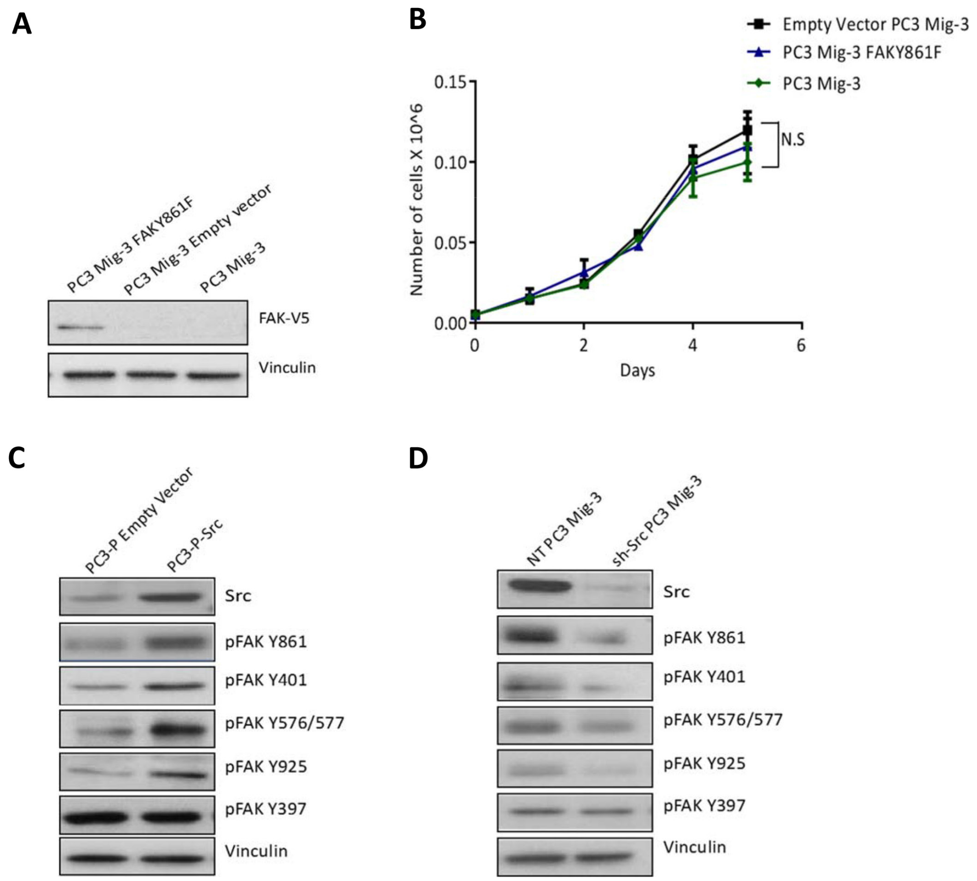

Supplementary Figure S3: Effects of FAKY861F expression on proliferation of PC3 Mig-3 cells and effect of Src and Yes on phosphorylation of individual tyrosine sites of FAK. A. Expression of FAK Y861F was determined by immunoblotting for the V-5 tag. **B.** Effect of expression of FAKY861F on proliferation of PC3 Mig-3 was determined by proliferation assay. N.S.-no statistical difference by Student's *t*-test. **C.** Src was overexpressed in PC3-P cells by transfection of the pCDNAIII-Src expression vector; empty vector was used as a control. Immunoblotting for Src and tyrosine phosphorylated FAK sites were performed. **D.** Src was silenced using by transfection with shSrc-GFP-puromycin (pGFP-V-RS). The vector expressing a Non targeting (NT) sequence was used as control. Immunoblotting for specific tyrosine phosphorylated FAK sites was performed.

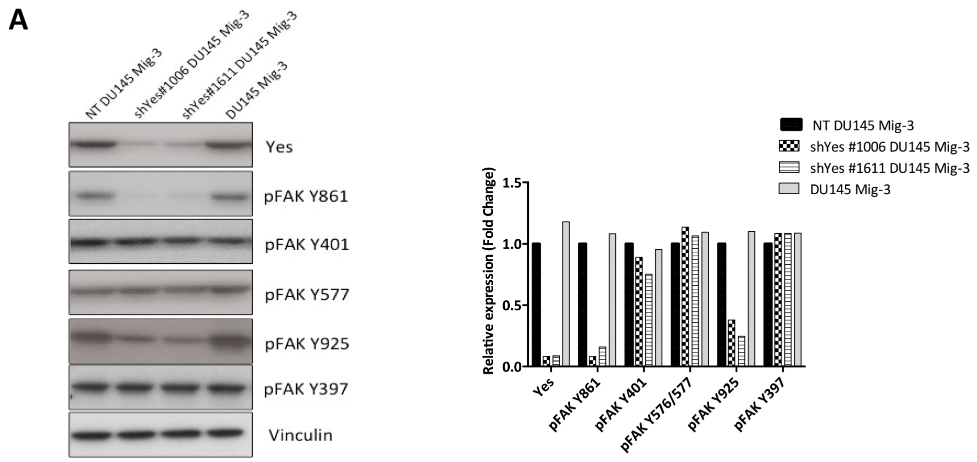

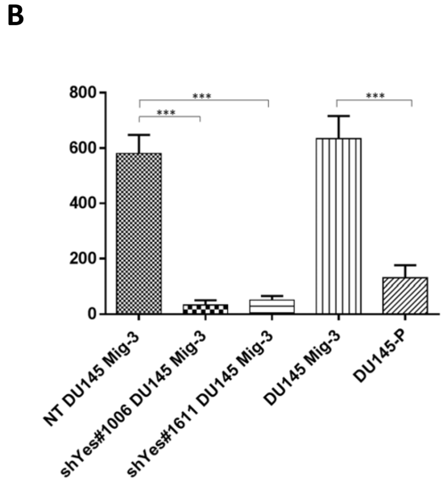

Supplementary Figure S4: Yes regulates phosphorylation of FAK Y861 and migration of DU145 Mig-3 cells. A. Knockdown of Yes using two shRNA sequences in DU145 Mig-3 cells and immunoblotting for specific tyrosine phosphorylated FAK sites. Quantification of phosphorylation of FAK at individual tyrosine sites normalized to vinculin. **B.** Effect of Yes knockdown on migration of DU145 Mig-3 cells. Bars represent means ± SD (lower panel). ***$p < 0.0001$ by Student's t-test of three independent assays performed in triplicate.

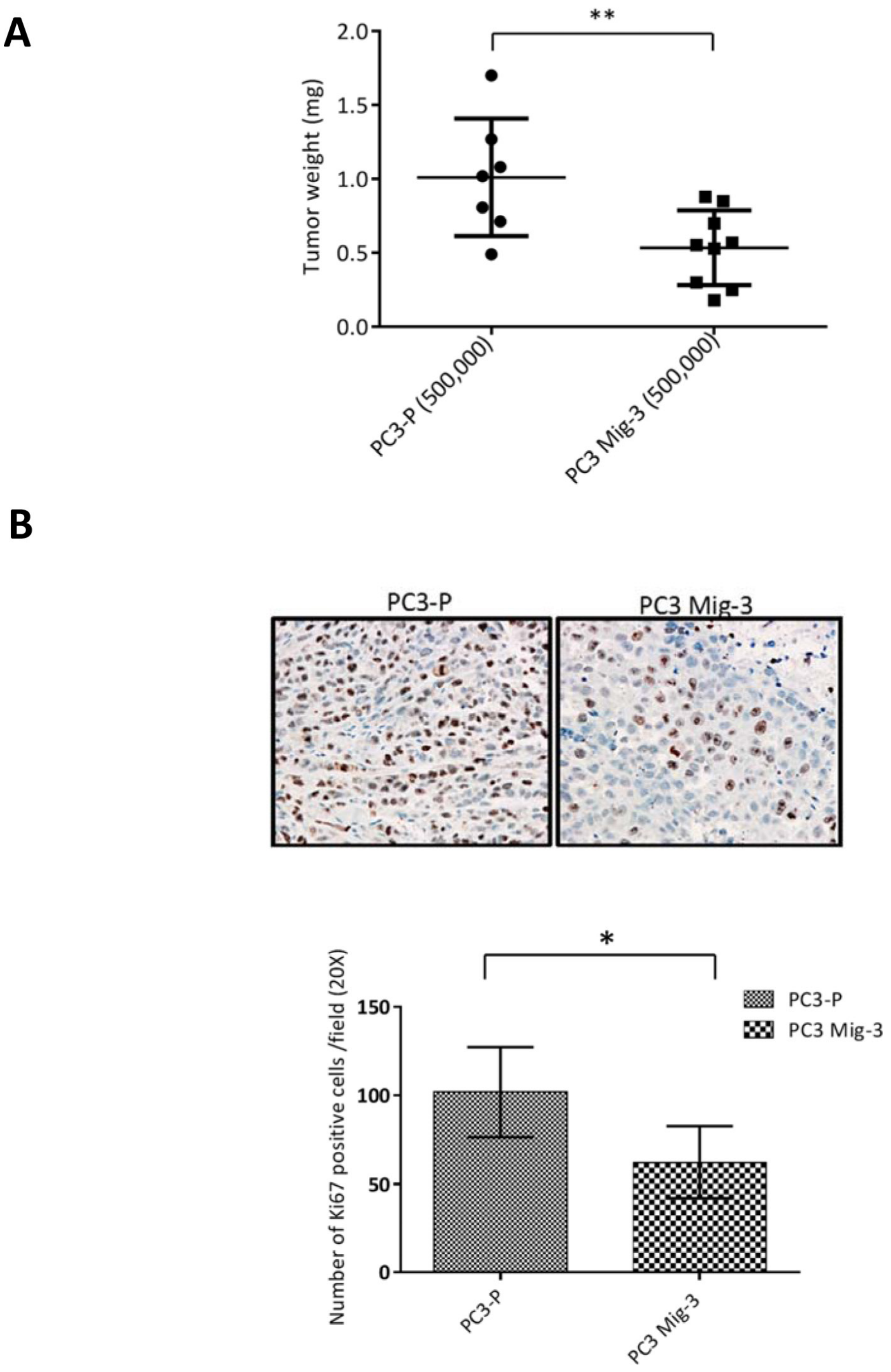

Supplementary Figure S5: PC3 Mig-3 cells form smaller tumors with reduced Ki67 staining relative to the parental cells. A. *In vivo* tumorigenicity of PCa cells was determined after intraprostatic injection of PC3-P cells and PC3 Mig-3 cells (6 mice/group). Graph represents average tumor weight ± SEM from PC3-P and PC3 Mig-3 tumors. $^{**}p < 0.001$ by Tukey's test. **B.** *In vivo* proliferation of PC3-P and PC3 Mig-3 was determined using Ki67 staining of tumor sections. Top left panel-PC3-P cells; top right panel-PC3 Mig-3 cells. Bars represent mean ± SD (bottom panel). $^{*}p < 0.05$ by Student's *t*-test.

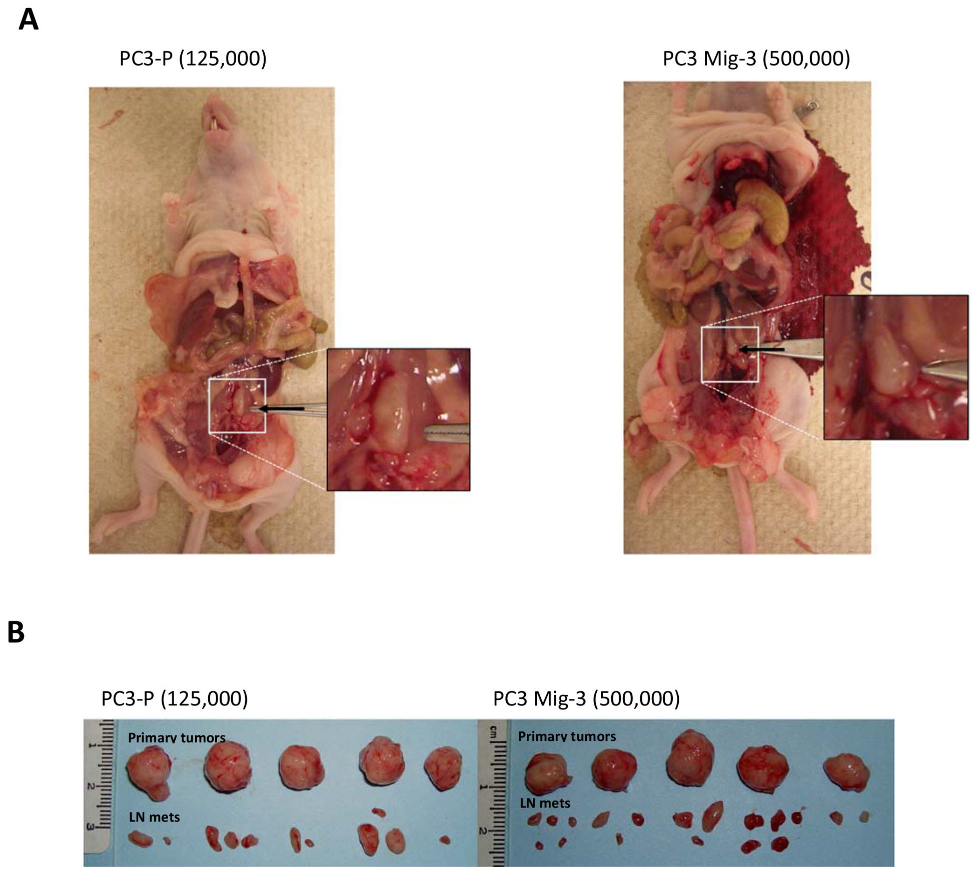

Supplementary Figure S6: Images of lymph node metastases and primary tumors after intraprostatic injection of PC3 cells. A. Images of LN mets from PC3-P and PC3 Mig-3 cells before dissection, arrows indicating visible lymph node metastases, inset, magnification of LN mets (20X). **B.** Images of primary tumors and lymph node metastases (LN mets) from PC3-P and PC3 Mig-3 cells isolated after intraprostatic injection of PC3 Mig-3 cells (6 mice/group) after dissection.

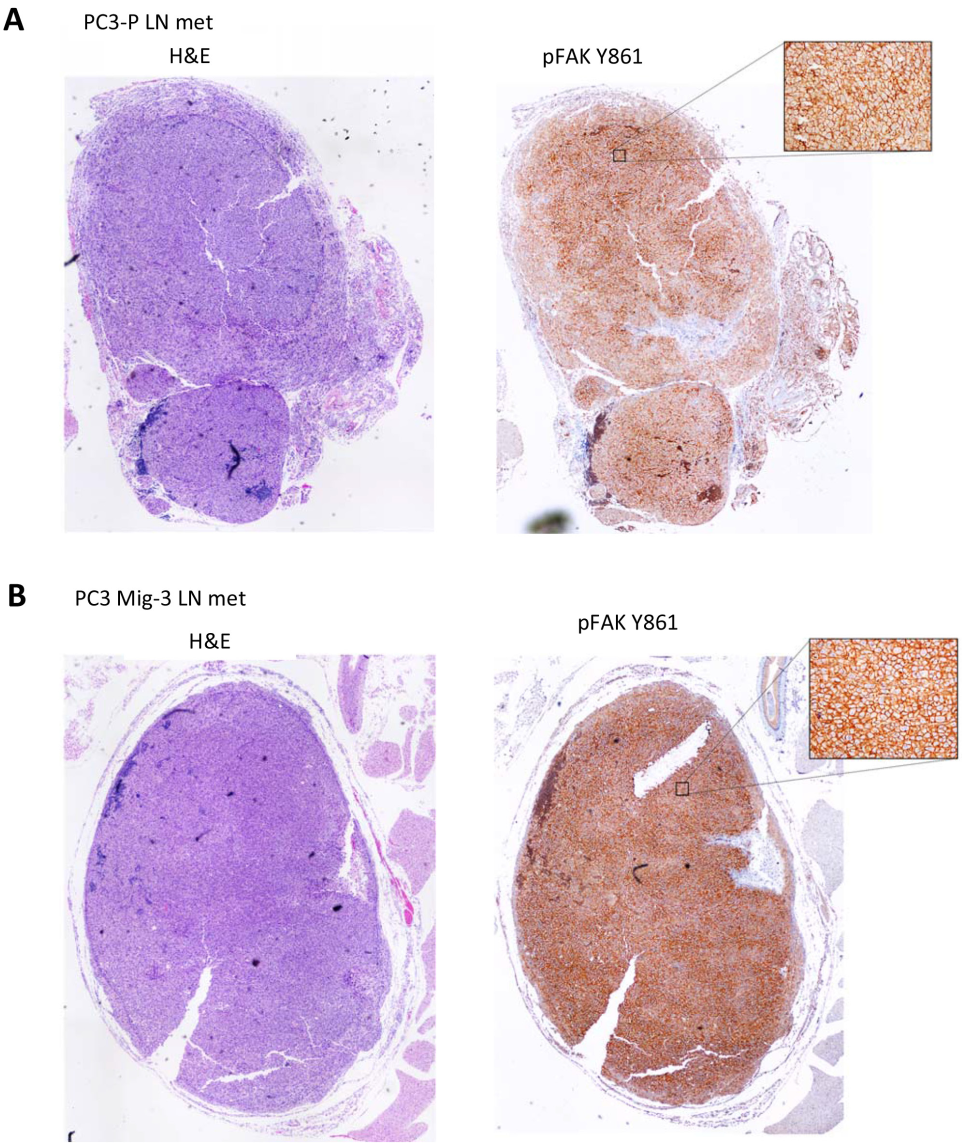

Supplementary Figure S7: PC3 Mig-3 cells form increased LN mets relative to parental cells. A. H&E staining on whole lymph node metastasis from PC3-P cells (Left panel), IHC indicating pFAK Y861 expression in the whole lymph node metastasis from PC3-P tumors (inset, magnification 20X) (Right panel). **B.** H&E staining on whole lymph node metastasis from PC3 Mig-3 cells (Left panel), IHC indicating pFAK Y861 expression in the whole lymph node metastasis from PC3-P tumors (inset, magnification 20X) (Right panel).

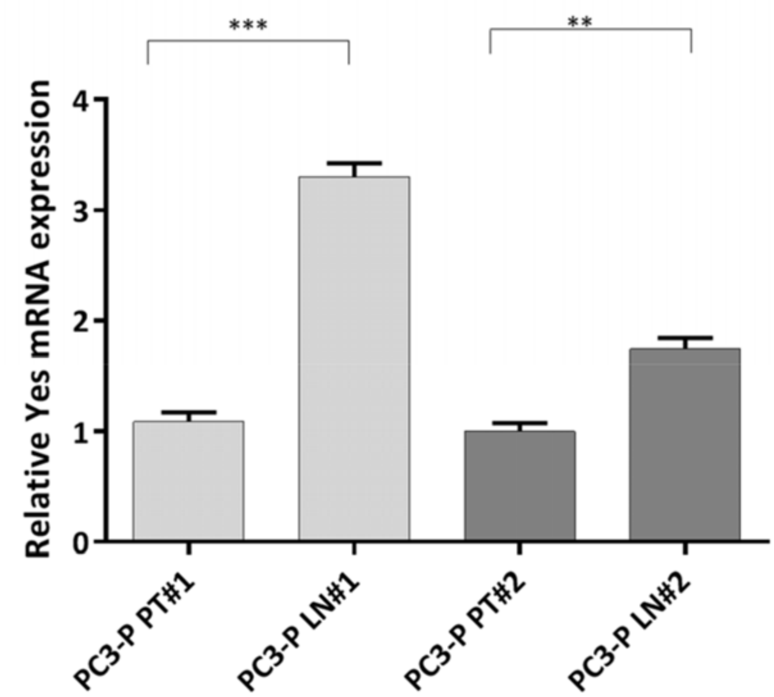

Supplementary Figure S8: Yes RNA expression in PC3-P primary tumors and lymph node metastases. qRT-PCR for Yes expression in PC3-P PT#1 (Primary tumor Mouse #1), PC3-P LN #1 (Lymph node met Mouse#1), PC3-P PT#2 (Primary tumor Mouse #2), PC3-P LN #2 (Lymph node met Mouse#2). ** $p < 0.001$, *** $p < 0.0001$.

Supplementary Table S1. List of antibodies

Antibody	Catalog#	Company
pFAK Y861	44-626G	Life Technologies
pFAK Y401	44-650G	Life Technologies
pFAK Y397	44-624G	Life Technologies
pFAK Y576	44-615G	Life Technologies
pFAK Y577	44-652G	Life Technologies
pFAK Y925	A14010	Life Technologies
pSrc Y416	2101	Cell Signaling Technologies
FAK	610088	BD Transduction
Src	2108	Cell Signaling Technologies
Yes	sc-46674	Santa Cruz Biotechnology
Fyn	sc-365915	Santa Cruz Biotechnology
Lyn	sc-376100	Santa Cruz Biotechnology
Axl	4977	Cell Signaling Technologies
Met	sc-162	Santa Cruz Biotechnology

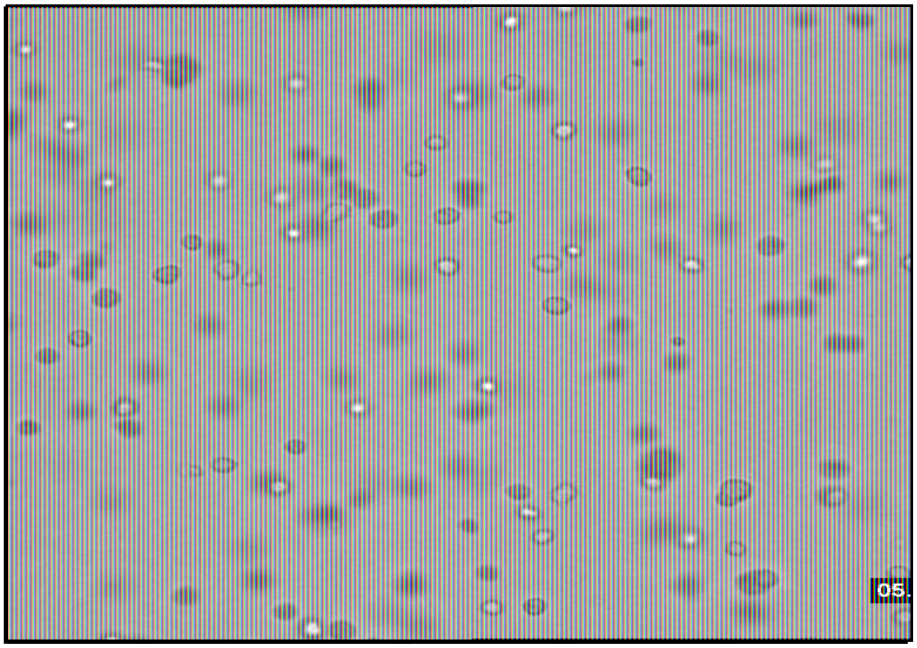

Supplementary Video 1:

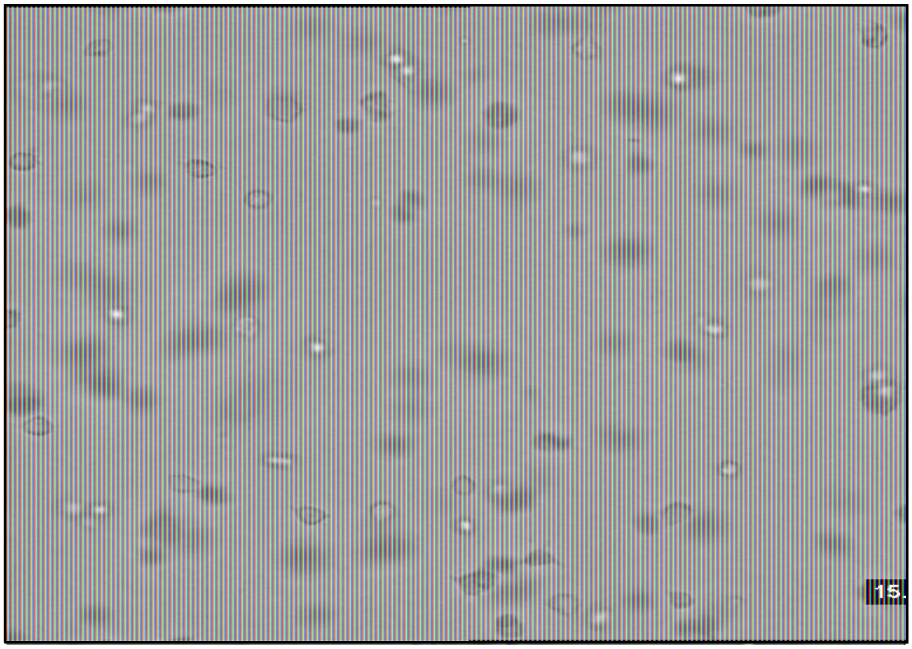

Supplementary Video 2:
